# Prediction of the effects of small molecules on the gut microbiome using machine learning method integrating with optimal molecular features

**DOI:** 10.1186/s12859-023-05455-1

**Published:** 2023-09-12

**Authors:** Binyou Wang, Jianmin Guo, Xiaofeng Liu, Yang Yu, Jianming Wu, Yiwei Wang

**Affiliations:** 1 School of Basic Medical Sciences, Southwest Medical University, Luzhou, 646000 China; 2 School of Pharmacy, Southwest Medical University, Luzhou, 646000 China; 3 Key Laboratory of Medical Electrophysiology, Ministry of Education and Medical Electrophysiological Key Laboratory of Sichuan Province, Institute of Cardiovascular Research, Southwest Medical University, Luzhou, 646000 China; 4 Sichuan Key Medical Laboratory of New Drug Discovery and Druggability Evaluation, Luzhou Key Laboratory of Activity Screening and Druggability Evaluation for Chinese Materia Medica, School of Pharmacy, Southwest Medical University, Luzhou, 646000 China

**Keywords:** Human gut microbiome, Anti-commensal effect, Machine learning, Molecular features, Consensus model

## Abstract

**Background:**

The human gut microbiome (HGM), consisting of trillions of microorganisms, is crucial to human health. Adverse drug use is one of the most important causes of HGM disorder. Thus, it is necessary to identify drugs or compounds with anti-commensal effects on HGM in the early drug discovery stage. This study proposes a novel anti-commensal effects classification using a machine learning method and optimal molecular features. To improve the prediction performance, we explored combinations of six fingerprints and three descriptors to filter the best characterization as molecular features.

**Results:**

The final consensus model based on optimal features yielded the F1-score of 0.725 ± 0.014, ACC of 82.9 ± 0.7%, and AUC of 0.791 ± 0.009 for five-fold cross-validation. In addition, this novel model outperformed the prior studies by using the same algorithm. Furthermore, the important chemical descriptors and misclassified anti-commensal compounds are analyzed to better understand and interpret the model. Finally, seven structural alerts responsible for the chemical anti-commensal effect are identified, implying valuable information for drug design.

**Conclusion:**

Our study would be a promising tool for screening anti-commensal compounds in the early stage of drug discovery and assessing the potential risks of these drugs in vivo.

**Supplementary Information:**

The online version contains supplementary material available at 10.1186/s12859-023-05455-1.

## Introduction

The human gut microbiota (HGM) consists of trillions of bacteria, archaea, phages, eukaryotic viruses, and fungi [1–3]. Through co-evolution, gut microbes have formed a good symbiotic relationship with humans. HGM uses the host environment and nutrition, but in return, provides many key functions to the body, such as synthesis of essential vitamins, removal of toxins, digestion of food, protection of intestinal mucosa, and immune regulation [3–6]. HGM is symbiotic with the host and maintains normal physiological processes in a state of dynamic equilibrium. There is growing evidence that the mechanisms of various diseases are associated with dysbiosis of HGM, including cardiovascular diseases, metabolic diseases, neurodegenerative diseases, and gastrointestinal diseases [7–9]. Thus, maintaining the ecological balance of HGM is crucial to human health. The function and composition of HGM can be influenced by various factors, including age, diet, host genetics, and medications [10]. Among these factors, medication drugs are considered one of the most important factors affecting the intestinal microbiota. Not only antibiotics targeting microorganisms, but also non-antibiotic drugs can have an impact on the composition and function of HGM [11, 12]. To systematically map interactions between drugs and HGM, high throughput in vitro study of more than 1000 drugs by Maier et al. revealed that one-quarter of drugs analyzed inhibited the growth of at least one of 40 representative intestinal bacterial strains [13]. The anti-commensal effect of such drugs can cause dysbiosis, which not only endangers human health but also reduces drug efficacy. However, as a new toxicity endpoint, the anti-commensal effects of drugs are not routinely tested in the current drug development process. Hence, paying more attention to the discrimination of the potential compounds with anti-commensal effects in the early stages of drug development is required.

Traditionally, the gut microbiome effects of drugs are detected experimentally. The anti-commensal or commensal effect of a drug is generally monitored by culturing the strain in vitro and measuring the change in optical density over time to monitor the effect of the drug on the growth of the colony [13–15]. However, the current experimental assays of the anti-commensal effect are time-consuming and labor-intensive, implying that testing all the chemicals on experimental platforms is impossible. In addition, the preconditions for the use of these experimental techniques are that the chemical compounds have been synthesized and are available in hand, which are not suitable for the fast development of virtual high-throughput screening nowadays. An alternative strategy is to use in silico methods to predict the anti-commensal effect of chemicals. Compared to detecting anti-commensal effect by laboratory tests, predicting this risk by in silico models is more time-saving and low-cost. Also, it does not involve any of the aforementioned preconditions.

To date, there are merely two computation prediction models for identifying the effect of drugs on HGM. In 2018, Zheng and coworkers established the first machine learning-based consensus classification model for the prediction of anti-commensal compounds, and their model provided an F1-score of 0.687 ± 0.023 on the test set [16]. In 2021, McCoubrey et al. [17] developed a machine learning model to predict whether drugs will impair the growth of 40 gut bacterial strains. Their best model gave AUC and F1-score of 0.857 ± 0.014 and 0.666 ± 0.042, respectively. Apparently, the machine learning models for predicting the effect of drugs on HGM are still rare, and their predictive performance in discriminating anti-commensal compounds from commensal compounds is limited. A gap remains for improving the predictive models of anti-commensal compounds.

**Fig. 1:**
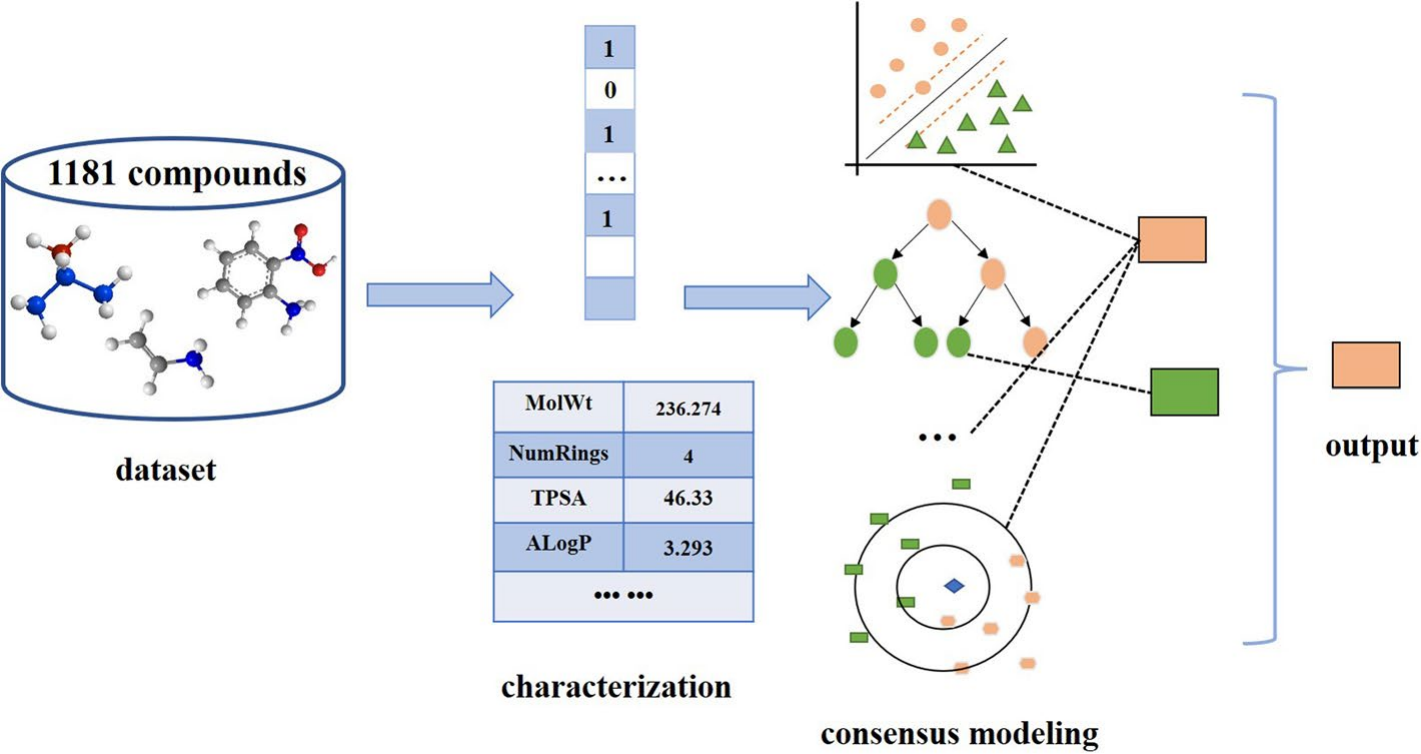
Flow chart for the development of consensus model for predicting commensal or anti-commensal compounds

In this study, we investigated a novel classification model for the prediction of commensal or anti-commensal compounds impacting HGM using six machine learning methods with an optimal set of molecular features. We applied six fingerprints, three sets of descriptors, and their combinations to extract the optimal set of molecular features for modeling to improve the prediction performance. The optimal novel consensus model was established and evaluated by internal and external validation. Furthermore, the important chemical descriptors, misclassified compounds, and the applicability domain (AD) of the best model were investigated to understand and interpret the model. Finally, structural alerts (SAs) of anti-commensal toxicity were carefully analyzed. The experimental procedure is shown in Fig. 1.

## Materials and methods

### Data preparation

To build different classification models and compare their performance, the same dataset used by Zhang’s group was adopted in this study [16]. All compounds in the dataset were collected from the single work of Maier et al. [13]. A compound is defined as an anti-commensal compound if it inhibits at least one of the bacteria in the human gut, while a compound is considered a commensal if it fails to inhibit any of the 40 typical bacteria in the experimental assay. The raw data were preprocessed through the following steps. First, for disconnected structures, only the organic fragments will be retained; Second, only compounds with common elements will be considered. The detailed process can be found in Zhang et al.'s research. After data cleaning, the dataset containing 1,181 diverse chemicals included 391 anti-commensal and 790 commensal compounds. We then randomly divided the entire dataset into a training set and external validation set according to the ratio of 8:2. To reduce the bias from the random splitting of the dataset, the entire set was randomly split 18 times. As a result, we obtained 18 randomly distributed data subsets consisting of training and external validation sets. The details of each data set are shown in Additional file 1: Table S1.

### Generation of molecular features

Algorithms are often thought of as the most important component of predictive model in drug discovery research. However, a dataset with a set of comprehensive, clean information for the structural, biological, and physical properties can drive an algorithmic approach to assessing potential drug candidates. Previous studies demonstrated that additional accuracy gains can be achieved when researchers customize the suitable features for each individual algorithmic application [18–21]. In order to obtain accurate features for the molecular structures, six types of molecular fingerprints were employed: MDL Molecular Access fingerprint (MACCS), PubChem fingerprint (PubChem), and four extended-connectivity fingerprints (ECFP4-1, ECFP4-2, ECFP6-1, ECFP6-2). These chosen fingerprints have been widely used to represent structural features of molecules for developing various classification model in drug discovery and yielde excellent prediction performances [22–25]. The names and dimensions of these fingerprints are summarized in Additional file 1: Table S2. MACCS and PubChem fingerprints were calculated using PaDEL-Descriptor software (version 2.21) [26]. ECFP was calculated by Python RDKit package version 2017.09. Moreover, to characterize molecules more accurately, three sets of molecular descriptors (MD), including 13MD, RDKit MD (RDMD) and Chemical Checker (CCMD), were calculated to describe the biological and physical properties of the chemicals. The detailed descriptions of these descriptors can be found in the corresponding literature [27–29]. The 13MD was calculated by Discovery Studio 3.1. The RDMD and CCMD were calculated using Descriptors module of Python RDKit package version 2017.09 (https://github.com/rdkit/rdkit) and Signaturizer package version 1.1.10 in Python (http://gitlabsbnb.irbbarcelona.org/packages/signaturizer), respectively. Since the values of different descriptors significantly span different ranges of values, their values were scaled to the same range (0, 1) by using the following formula:

$${x}^{*}= \frac{x-{x}_{min}}{{x}_{max}-{x}_{min}}$$

where* x* is the original value, x* is the normalized value, and *x*_*max*_ and *x*_*min*_ are the maximum and minimum values of a descriptor, respectively. The min–max scaler is commonly used for data scaling in many classification problems [30–32]. For the datasets in this work, this method achieved better or comparable performance to the standard scaler (Additional file 1: Table S3).

### Prediction models based on six machine learning algorithms

In the present study, six classical machine learning algorithms, including support vector machine (SVM) [33], *k*-nearest neighbor (*k*‑NN) [34], random forest (RF) [35], naive Bayes (NB) [36], Gradient boosting machine (GBM) [37], and extreme gradient boosting (XGBoost) [38] were used to construct the prediction models for the anti-commensal effects. Detailed descriptions of these methods can be found in the Additional file 1: Supporting Method. Six types of molecular fingerprints and their combination with three sets of descriptors, a total of 24 molecular characterization sets were obtained to represent the structural information of the compounds. Each algorithm was modeled with the 24 sets of molecular features, followed by a hyperparameterization. SVM models were built by the radial basis kernel (RBF) function. The regularization parameter C and the kernel parameter gamma were also optimized. *k*-NN used the weighting schemes and the number of neighbors for the optimization parameters. For RF, the best split was calculated based on the number of trees in the forest. GBM was tuned using the learning rate parameters and the number of decision trees. For XGB, the parameter of the maximum depth of a tree and the minimum sum of the instance weight needed in a child were optimized. All the key parameters for each method were listed in Additional file 1: Table S4, and the default values were used in other parameters not mentioned. In order to obtain the best model for the 24 molecular features with the best parameters, these parameters were fine-tuned using a five-fold cross-validation method and repeated ten times to reduce random variance and for robust performance. SVM, *k*-NN, RF, NB, GBM, and XGB models were implemented in the *scikit-learn* package of Python (version 0.23.2) [39]. The XGB model was built using *XGBoost* package version 1.4.2 in Python (https://github.com/dmlc/xgboost).

Finally, we obtained the most optimal model for predicting the anti-commensal compounds from the six algorithms via a voting method. That is, the machine learning algorithm that produces the largest ratio of the best models based on the 24 molecular features for the 18 datasets is considered the best one.

### Consensus modeling

Consensus modeling is the combination of predictions from multiple member models to form a consensus result [40]. Compared with an individual model, the consensus model can benefit from various representations of the chemical structures and fits more molecular features [40–42]. In the process of predicting the ADMET (absorption, distribution, metabolism, excretion, and toxicity) properties of compounds, the consensus model tends to have higher predictive accuracy, is more reliable, and is more generalizable. Meanwhile, to make a parallel comparison with the reported HGM model, a consensus model is established based on the methodology of this study. On the basis of the machine learning algorithm selected in Sect. “Prediction models based on six machine learning algorithms”, this machine learning algorithm was applied with the 24 features to build the model. The optimal molecular features were also selected by using the voting method. Finally, we obtained the best combination of the machine learning method and molecular features. For each of the 18 datasets, the best model based on the optimal combination of the machine learning method and molecular features was established. Ultimately, the consensus model establishes by simply averaging the values of the 18 single models.

### Performance evaluation

The following six indicators were used to estimate the predictive performance of all the models: sensitivity (SE), specificity (SP), accuracy (ACC), Matthew’s correlation coefficient (MCC), F1-score and area under the curve (AUC). These indicators detailed formulas are shown below:

$$ACC=\frac{TP+TN}{TP+FP+TN+FN}$$

$$SE=\frac{TP}{TP+FP}$$

$$SP=\frac{TN}{TN+FN}$$

$$MCC=\frac{TP\times TN-FP\times FN}{\sqrt{(FP+TN)(FP+TP)(FN+TN)(FN+TP)}}$$

$$F1-score=\frac{2TP}{(2TP+FN+FN)}$$

where TP is the number of anti-commensal compounds that are predicted correctly, TN is the number of commensal compounds that are predicted correctly, FP is the count of commensal compounds that are incorrectly predicted as anti-commensal compounds, and FN is the count of anti-commensal compounds that are incorrectly predicted as commensal compounds.

SE and SP, indicate the predictive ability of the model for anti-commensal drugs and commensal drugs, respectively. ACC illustrates the model's ability to predict the overall data. AUC denotes the area under the receiver operating characteristic (ROC) curve, which is a comprehensive evaluation index for the overall predictive performance of the model. F1-score is the harmonic mean of recall and precision. Even when the data set was extremely unbalanced, F1-score could still indicate the overall classification performance of the model. It is worth mentioning that the AUC value is often used to evaluate the predictive ability of ADMET classifiers. Therefore, we employed F1-score as well as AUC value to measure the quality of the binary classification and adopted them as the criteria to select the best model.

### Applicability domain analysis

The definition of AD is an important consideration for structure‐activity relationship (SAR) modeling according to the OECD guidelines [23]. The AD of the prediction models means that the model prediction is reliable in this chemical space region. For classification models, the distance-based method is commonly used to define AD [43]. In this study, the Euclidean distance method was applied to identify the AD of the prediction models. The method is to compare the Euclidean distances between the compounds and the dataset with a predefined threshold. In the present study, the structures of the compounds were characterized by the optimal molecular features, and then the Euclidean distance between the test set and the training set was calculated. This analysis was conducted by the AMBIT Discovery software (version 0.04) (http://ambit.sourceforge.net) with the threshold set at 99%. A more detailed description of the Euclidean distance-based approach can be found in the literature [43].

### Structural alerts analysis

The structural alerts (SAs) refer to the key substructures that cause the toxicity of compounds. In order to better evaluate typical structural fragment related to anti-commensal effects, SAs were analyzed by the information gain (IG) method coupled with structural fragments frequency analysis. The detailed definitions of IG and the frequency of a fragment are listed in the Additional file 1: Supplementary Formula. In the present study, the substructure fragments of all compounds were derived from Klekota–Roth fingerprints (KRFP) [44]. If a substructure with a high IG value were to be presented more frequently in anti-commensal compounds than commensal compounds, this substructure would be regarded as an SA for the anti-commensal effects [45].

## Results and discussion

### Optimal machine learning algorithm selection

To rigorously select the suitable machine learning algorithm for predicting the anti-commensal effect of drugs, six classical and commonly used machine learning algorithms (SVM, *k*-NN, RF, NB, GBM, and XGB) were employed and compared directly. Twenty-four sets of different molecular features were generated for all 391 anti-commensal compounds and 790 commensal compounds in the dataset. The performance of each model for predicting the anti-commensal chemicals was assessed by five-fold cross-validation on 18 groups of training datasets. The F1-score and AUC values of the best models developed by each machine learning algorithm integrating with 24 sets of molecular features were summarized in Additional file 2: Tables S5 and S6, respectively. We found that the trend of AUC was in accordance with that of F1 score, thus the F1 values of each model were mainly analyzed in the following work. The XGB algorithm provided the best performance in most cases, either on different molecular characterization sets or different data groups. Table 1 lists the ratio of each algorithm that achieved the best predictive result by using 24 sets of molecular features. For the 24 molecular features, the XGB algorithm gave the largest ratio of the highest F1-scores (larger than 11/24) on each group of the training dataset. Moreover, we performed a statistical analysis of the optimal F1-score of the XGB models with the of other models (SVM, *k*-NN, RF, NB and GBM), and *p*-value were 3.44 × 10^−13^, 2.31 × 10^−16^, 3.03 × 10^−4^, ok3.94 × 10^−18^ and 7.53 × 10^−6^, respectively. It is indicated that the results differed in significance. These results suggest that the XGB algorithm has a better ability to discriminate positives from negatives than other algorithms. Thus, we selected the XGB algorithm as the optimal one for further analysis.

**Table 1:** The ratio of each algorithm achieving the best performance based on the 24 sets of molecular features

	XGB	SVM	*k*-NN	RF	NB	GBM
1	14/24	7/24	2/24	0/24	0/24	1/24
2	11/24	8/24	1/24	0/24	0/24	4/24
3	14/24	7/24	0/24	1/24	0/24	2/24
4	13/24	7/24	2/24	1/24	0/24	1/24
5	14/24	7/24	0/24	0/24	0/24	3/24
6	15/24	8/24	0/24	0/24	0/24	1/24
7	16/24	5/24	1/24	0/24	0/24	2/24
8	14/24	9/24	0/24	0/24	0/24	1/24
9	12/24	8/24	0/24	0/24	2/24	2/24
10	15/24	4/24	1/24	1/24	0/24	3/24
11	15/24	8/24	1/24	0/24	0/24	0/24
12	15/24	5/24	1/24	0/24	0/24	3/24
13	12/24	7/24	0/24	2/24	0/24	3/24
14	16/24	4/24	2/24	1/24	0/24	1/24
15	16/24	6/24	1/24	1/24	0/24	0/24
16	11/24	10/24	0/24	2/24	0/24	1/24
17	13/24	8/24	1/24	0/24	0/24	2/24
18	14/24	6/24	0/24	1/24	0/24	3/24

### Molecular descriptors optimization

To obtain the optimal molecular features for characterizing the structure and properties of the anti-commensal/commensal compounds, 24 sets of molecular features, including six types of fingerprints and 18 combinations of molecular fingerprints and descriptors, were used to establish a model based on the XGB algorithm. The detailed predictive results of the top XGB models with 24 sets of molecular features on each training set by five-fold cross-validation are presented in Table 2. From Table 2, the mean values of F1-score, ACC, and AUC for the top XGB models were in the range of 0.696–0.742, 81.7–80.4%, and 0.779–0.806, respectively. It indicated that all the top XGB models had a high prediction precision and recall rate. Table 2 clearly shows that all the top XGB models were described by the combinations of molecular fingerprints and descriptors. Meanwhile, we found that the models using the MACCS + 13MD feature set occupied 16 of the 18 sets of optimal models based on the XGB algorithm. Therefore, the XGB models using the combinations of MACCS molecular fingerprints with 13MD had better performance than models using the other 23 sets of molecular features.

**Table 2:** Five-fold cross-validation results of the top classification model from each training set based on XGB

Group	Features	SE (%)	SP (%)	ACC (%)	MCC	AUC	F1-score
1	13MD + MACCS	68.2 ± 1.4	91.7 ± 0.6	84.1 ± 0.7	0.627 ± 0.019	0.800 ± 0.009	0.734 ± 0.014
2	13MD + MACCS	63.9 ± 0.9	91.9 ± 0.5	83.0 ± 0.4	0.594 ± 0.010	0.779 ± 0.005	0.703 ± 0.008
3	13MD + MACCS	68.5 ± 1.1	89.4 ± 0.4	82.0 ± 0.5	0.598 ± 0.012	0.789 ± 0.006	0.727 ± 0.009
4	13MD + MACCS	63.8 ± 0.9	90.8 ± 0.7	82.0 ± 0.6	0.576 ± 0.014	0.773 ± 0.006	0.696 ± 0.009
5	13MD + MACCS	68.5 ± 1.8	91.0 ± 0.4	82.9 ± 0.7	0.620 ± 0.017	0.797 ± 0.010	0.739 ± 0.013
6	13MD + MACCS	66.1 ± 1.1	91.6 ± 0.8	83.0 ± 0.6	0.607 ± 0.014	0.788 ± 0.007	0.721 ± 0.010
7	13MD + MACCS	66.2 ± 1.3	90.2 ± 0.8	82.1 ± 0.9	0.588 ± 0.020	0.782 ± 0.010	0.712 ± 0.014
8	13MD + MACCS	69.0 ± 1.4	92.2 ± 0.7	84.3 ± 0.8	0.641 ± 0.018	0.806 ± 0.007	0.747 ± 0.013
9	13MD + MACCS	66.1 ± 1.5	89.7 ± 0.7	81.7 ± 0.5	0.581 ± 0.013	0.779 ± 0.007	0.708 ± 0.009
10	13MD + MACCS	69.2 ± 1.2	90.0 ± 0.7	82.5 ± 0.7	0.613 ± 0.017	0.796 ± 0.008	0.739 ± 0.011
11	13MD + PubChem	69.3 ± 1.7	91.1 ± 0.4	83.8 ± 0.6	0.628 ± 0.013	0.802 ± 0.008	0.738 ± 0.012
12	13MD + MACCS	68.4 ± 0.8	90.9 ± 0.6	83.2 ± 0.4	0.617 ± 0.009	0.797 ± 0.004	0.734 ± 0.006
13	13MD + MACCS	69.6 ± 1.8	90.5 ± 0.6	83.2 ± 0.7	0.622 ± 0.015	0.801 ± 0.009	0.742 ± 0.012
14	13MD + MACCS	67.6 ± 0.9	91.3 ± 0.7	83.2 ± 0.5	0.616 ± 0.010	0.794 ± 0.007	0.732 ± 0.005
15	13MD + MACCS	68.3 ± 1.2	90.5 ± 0.6	83.0 ± 0.5	0.611 ± 0.011	0.794 ± 0.006	0.729 ± 0.009
16	13MD + MACCS	68.7 ± 1.0	90.0 ± 0.8	82.8 ± 0.5	0.607 ± 0.010	0.793 ± 0.004	0.728 ± 0.006
17	13MD + MACCS	66.7 ± 0.9	90.9 ± 0.6	82.9 ± 0.4	0.601 ± 0.010	0.788 ± 0.005	0.716 ± 0.007
18	RDMD + PubChem	69.0 ± 1.5	91.6 ± 0.5	84.0 ± 0.7	0.631 ± 0.016	0.803 ± 0.009	0.741 ± 0.013

The three sets of molecular descriptors were used to generate models to further determine whether the models based on the combination of molecular representations and the optimal XGB algorithm are advantageous. Additional File 1: Table S7 displayed the F1-score values of the optimal XGB models for each training set based on the combined features (fingerprints integrated with descriptors), fingerprints, and descriptors, respectively. As shown in Additional file 1: Table S7, the mean F1-score value of all the top XGB models developed by the combined features was in the range of 0.696 ~ 0.747, and the corresponding training set described by fingerprints or molecular descriptors ranged from 0.648 to 0.720, suggesting that the models based on the combination features yielded better performance than other models based on fingerprints or molecular descriptors alone. In addition, we found that all the evaluation metrics of models using a combination of molecular features are higher than those of the models based on only descriptors for each training set. These results clearly demonstrate that the XGB models with a combination of optimal molecular fingerprints and descriptors provide a substantially improved predictive ability.

In general, the combination of MACCS and 13MD is the most optimal feature set to develop the prediction models for anti-commensal compounds. MACCS fingerprint is a substructure-based fingerprint that contains most atomic properties, topologies properties of chemical bonds, and atomic neighborhoods. 13MD contains 13 commonly used molecular properties such as molecular solubility and polarity. Thus, the essential information about molecular structure and molecular properties contained in the optimal features are closely related to the anti-commensal effect.

### Predictive performance of consensus model

The consensus modeling is to integrate several weak learners into one strong learner, which can improve the robustness and generalization capability of the SAR model. To further obtain the excellent consensus model, we used the XGB algorithm integrating with the optimal set of combination features (MACCS + 13MD) to train the 18 groups of training datasets by five-fold cross-validation. The detail predictive results of the top model for each group are outlined in Additional file 1: Table S8. From Additional file 1: Table S8, the mean value of the F1-score was in the range of 0.696–0.742. And the average value of ACC, the average value of SE, the average value of SP, the average value of MCC, and the average value of AUC ranged from 81.7 to 84. 3%, 63.8 to 69.6%, 89.4 to 91.9%, 0.576 to 0.641, and 0.773 to 0.806, respectively.

Based on the 18 individual models, a consensus model named 13MD-CM was established by simply averaging the values of the single models. As shown in Table 3, the consensus model provided relatively optimal results with an average SE of 67.4%, an average SP of 90.8%, an average ACC of 82.9%, an average MCC of 0.608, the average AUC of 0.791 and an average F1-score of 0.725 in internal validation. Furthermore, external validation was used to assess the capability of our consensus model. The consensus model yielded an ACC of 82.2 ± 2.6%, F1-score of 0.669 ± 0.05, SE of 60.7 ± 6.7%, SP of 91.1 ± 3.3%, MCC of 0.554 ± 0.058 and AUC of 0.759 ± 0.03 on the external validation dataset. From these results, it can be seen that the consensus model provides high prediction accuracy. In addition, we explored the reliability of the 13MD-CM, and the AD of the 18 individual models for building the 13MD-CM was defined. The defined AD covered all 18 training datasets with a value of 99.0%, and the average value covering the 18 external validation sets was 98.9% (Table 4). The majority of compounds in the dataset were in the AD, indicating that the predictive performance of the consensus model was quite plausible for the external validation sets.

**Table 3:** Results from the five-fold cross-validation and external validation of the 13MD-CM

	SE (%)	SP (%)	ACC (%)	MCC	AUC	F1-scorce
Five-fold cross-validation	67.4 ± 1.8	90.8 ± 0.8	82.9 ± 0.7	0.608 ± 0.017	0.791 ± 0.009	0.725 ± 0.014
External validation	60.7 ± 6.7	91.7 ± 3.3	82.2 ± 2.6	0.554 ± 0.058	0.759 ± 0.03	0.669 ± 0.05

**Table 4:** The number of drugs inside and outside of the AD

		Inside		Outside		AD coverage (%)
		P	N	P	N	
1	Training set	301	637	4	5	99.0
	External validation set	86	148	0	2	99.2
2	Training set	297	639	4	5	99.0
	External validation set	90	144	0	2	99.2
3	Training set	329	607	4	5	99.0
	External validation set	58	177	0	1	99.6
4	Training set	304	632	4	5	99.0
	External validation set	82	153	1	0	99.6
5	Training set	333	603	4	5	99.0
	External validation set	54	181	0	1	99.6
6	Training set	311	625	4	5	99.0
	External validation set	76	159	0	1	99.6
7	Training set	315	621	2	7	99.0
	External validation set	72	159	2	3	97.9
8	Training set	316	620	3	6	99.0
	External validation set	69	159	3	5	96.6
9	Training set	315	621	5	4	99.0
	External validation set	71	164	0	1	99.6
10	Training set	337	599	3	6	99.0
	External validation set	50	183	1	2	98.7
11	Training set	311	625	4	5	99.0
	External validation set	75	156	1	4	97.9
12	Training set	305	631	3	6	99.0
	External validation set	81	152	2	1	98.7
13	Training set	319	617	3	6	99.0
	External validation set	68	165	1	2	98.7
14	Training set	325	611	4	5	99.0
	External validation set	62	174	0	0	100
15	Training set	319	617	3	6	99.0
	External validation set	68	167	1	0	99.6
16	Training set	315	621	3	6	99.0
	External validation set	72	160	1	3	98.3
17	Training set	316	620	3	6	99.0
	External validation set	72	162	0	2	99.2
18	Training set	312	624	4	5	99.0
	External validation set	74	159	1	2	98.7

### Misclassified anti-commensal compounds analysis

**Fig. 2:**
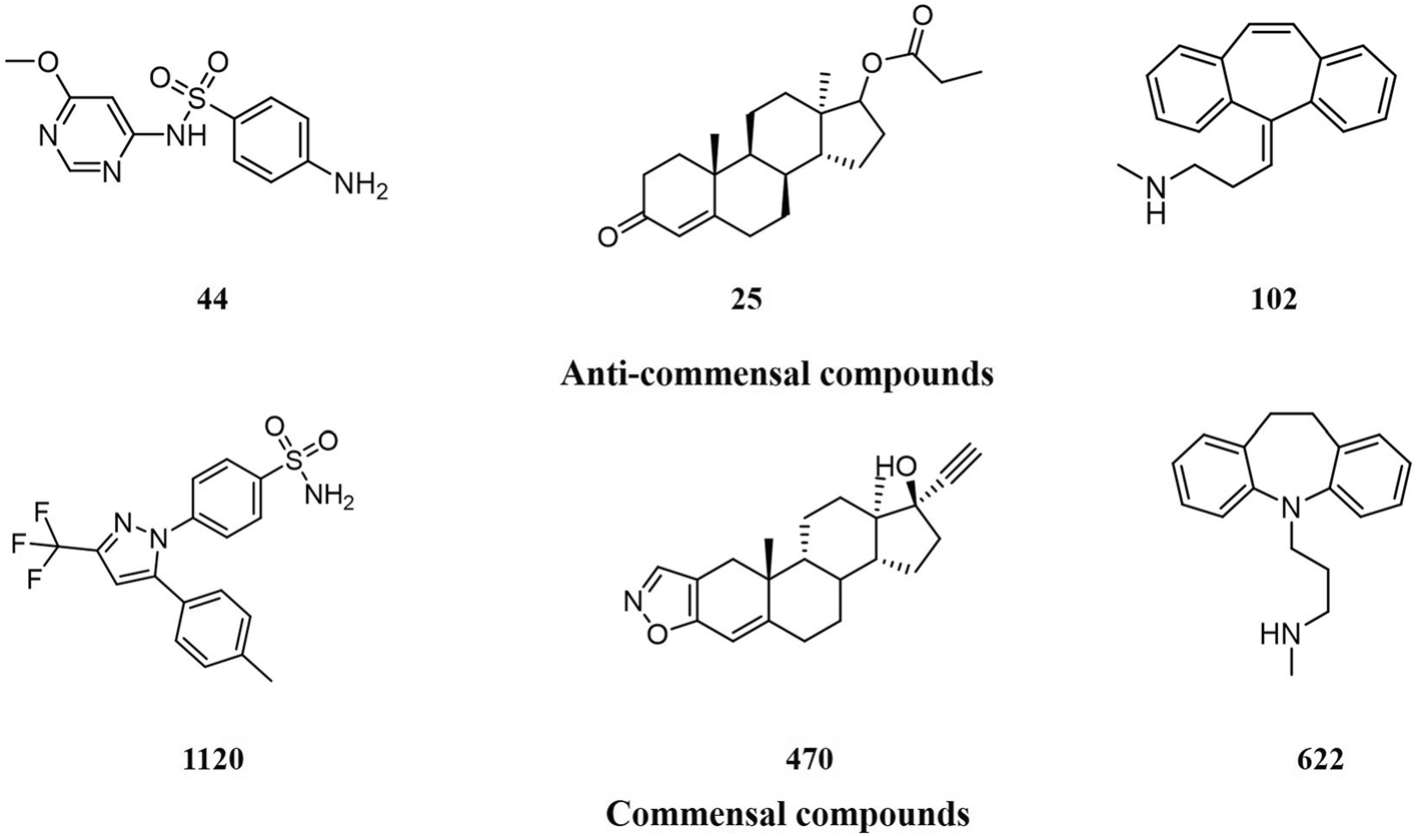
Structures of the six misclassified compounds in the external validation set

Although our consensus model achieved favorable results in overall accuracy on the external validation sets, the SE values were comparatively low. We analyzed the 20 anti-commensal compounds misclassified five times or more for the external validation set. The structures of all anti-commensal compounds that were misclassified more than five times are listed in Additional file 1: Fig. S1. As shown in Additional file 1: Fig. S1, seven out of 20 misclassified anti-commensal compounds contain stereospecific structures that can lead to compounds with radically different pharmacological properties. But the molecular fingerprints used in our study are impossible to accurately describe the entire structure of the compounds, especially the stereoisomers. Most misclassified compounds contain long carbon chains, and carbon chain isomers may exist in the gut microbial environment in vivo, resulting in different properties. However, the in vivo variation of agents was not considered in this research, so this could be the reason for the misclassification of these compounds. In addition, seven out of the 20 misclassified anti-commensal compounds, comprising more than three paracyclic and sulfamide were misclassified commensal agents. A careful analysis of these substructures revealed the presence of misclassified anti-commensal compounds and commensal compounds (Fig. 2), suggesting that chemicals with similar structures have completely opposite activities, leading to the misidentification of the model. It is also possible to reason that the dataset is unbalanced, with less than 50% of anti-commensal compounds, which may lead to biased predictions in favor of the larger-sized categories (SP of 91.1 ± 3.3%, SE of 60.7 ± 6.7%). More accurate molecular descriptors representing the structure and activity of agents, richer data, and more advanced algorithms should be applied in SAR modeling to address the above issues.

### Important descriptors analysis

**Fig. 3:**
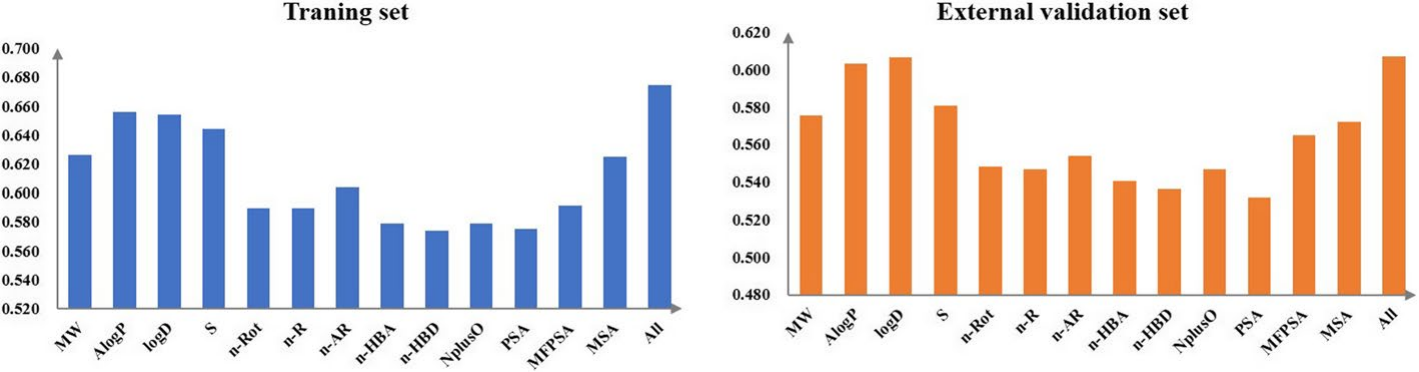
SE values of the single descriptor and full descriptors models

Individual descriptors from 13MD were used to establish the XGB model for each training dataset to investigate the important descriptors driving the performance of the models. The prediction models based on individual descriptors were evaluated by the five-fold cross-validation. Because the SE value is considered an essential indicator for assessing the ability of the model to identify positive compounds, the SE value was chosen as the criterion for selecting key descriptors. Figure 3 shows the average predictive ability of each descriptor from 13MD used for assessing the proposed XGB model. The summary plot indicates the relationship between the descriptor value and its impact on the model prediction. The importance order ranking of the models based on SE values is AlogP, logD, S, MW, MSA, n-AR, MFPSA, n-R, n-AR, n-HBA, NplusO, PSA, and n-HBD descriptor. We can obtain the contribution of each descriptor to the model prediction based on the order ranking. It is evident that the model based on AlogP offered the highest SE value of 65.6%, which was equal to the average SE values of the models with full descriptors. Based on the result, the AlogP descriptor may serve as the primary feature for anti-commensal compounds. Therefore, more attention to AlogP and its related properties is warranted to avoid the anti-commensal effects of the drugs.

### Comparison with previous predictors

We further carefully compared our model with various models reported in the literature. In 2018, Zhang’s group applied four machine learning methods (*k*-NN, SVM, GBM, and RF) with four types of ECFP to establish the first consensus model named CM for predicting anti-commensal effects [16]. In order to make a reasonable comparison with the model of Zhang et al., we adopted the same machine learning methods with our optimal molecular feature (13MD + MACCS) to develop a consensus model. Because detailed data groupings for Zhang were not available, we only compared the results of five-fold cross-validation on the training set for the sake of fairness. The statistical results for the 18 groups of datasets given by best models based on four machine learning methods with 13MD + MACCS are listed in Additional file 1: Table S9. As seen from Additional file 1: Table S9, the consensus model with 13MD + MACCS provided an F1-score of 0.716 ± 0.013, which was relatively higher than that of Zhang's work (0.681 ± 0.037). It suggested that the optimal molecular feature combination of MACCS and 13MD is more suitable for characterizing anti-commensal compounds than ECFP and further illustrated that molecular descriptors can improve the model's prediction ability.

### Identification of structural alerts

**Fig. 4:**
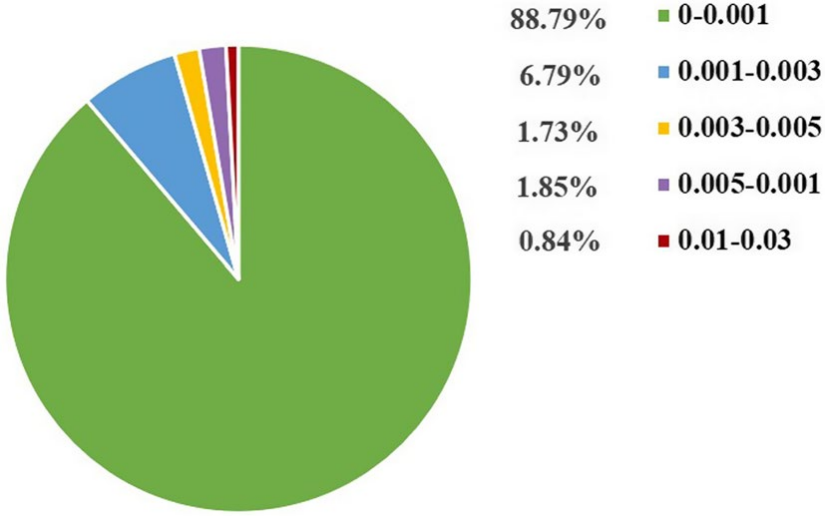
IG value distributions of the KRFP fragments

To define structural fragments of compounds relevant to the anti-commensal effects, we analyzed the structural fragments of anti-commensal compounds which appeared more than ten times in the dataset. The distribution of IG values for each fragment is shown in Fig. 4. From the results, it can be concluded that the IG values of all the 4,860 fragments ranging from 0 to 0.027, and the IG values of very few fragments were above 0.01. According to the values of IG and frequencies of fragments, seven SAs and representative anti-commensal compounds were achieved. As shown in Table 5, the No.1 and No.3 structures are commonly found in amide and quinolone antibiotics, respectively. And the threat of antibiotics to human gut microbes has been extensively described in the literature. Among these seven substructures, both phenothiazine (No.6) and imidazole ring (No.8) are essential nitrogen-containing heterocyclic structures widely used in anti-psychotic and antifungal drugs, respectively. Therefore, the anti-commensal effect of these three classes of drugs should be of concern in clinical applications. Generally, these substructures of high IG values appeared far more frequently in anti-commensal compounds than non-anti-commensal compounds. A compound containing one or more such substructures tend to possess a higher percentage of the anti-commensal property.

**Table 5:** Seven SAs of anti-commensal effect and their representative structures

No	Structure	IG	Freq_P	Freq_N	Representative structure
1		0.0199	2.52	0.25	
2		0.0152	3.02	0	
3		0.0132	3.02	0	
4		0.0114	2.40	0.31	
5		0.0097	2.67	0.18	
6		0.0094	2.16	0.43	
7		0.0087	2.79	0.11	

## Conclusion

This study established a novel and powerful consensus model (13MD-CM) for predicting the anti-commensal effect by the optimal set of molecular features (MACCS + 13MD). A series of cross-validation and external validations corroborated our model's significant effectiveness and promising performance, especially in correctly identifying anti-commensal compounds. The interpretability of the 13MD-CM model was analyzed by important descriptors and misclassified compounds. AlogP was deemed the most important descriptor for the model's performance. Finally, seven SAs about the anti-commensal effect were obtained. In summary, our research has uncovered a reliable and robust classification consensus model for predicting the chemical anti-commensal effect and provided key substructures for risk assessment of the anti-commensal property. These results would be helpful for effectively assessing the anti-commensal effect during the early drug development stage.

### Supplementary Information

**Additional file 1**. Detailed information of supplementary methods, supplementary formula, supplementary tables S1–S4 and supplementary tables S7–S9, supplementary figure S1.

**Additional file 2**. Detailed information of supplementary tables S5–S6.

## Data Availability

The data presented in this study are available in the Supplementary Materials of the paper.

## Abbreviations

HGM: Human gut microbiome
AD: Applicability domain
SAs: Structural alerts
MACCS: MDL Molecular Access fingerprint
PubChem: PubChem fingerprint
ECFP: Extended-connectivity fingerprints
MD: Molecular descriptors
RDMD: RDKit molecular descriptors
CCMD: Chemical Checker
SVM: Support vector machine
*k*‑NN: *k*-Nearest neighbor
RF: Random forest
NB: Naive bayes
GBM: Gradient boosting machine
XGBoost: Extreme gradient boosting
RBF: Radial basis kernel
SE: Sensitivity
SP: Specificity
ACC: Accuracy
MCC: Matthew’s correlation coefficient
AUC: Area under the curve
KRFP: Klekota–Roth fingerprints
