# Supplementary material for: Prediction of the effects of small molecules on the gut microbiome using machine learning method integrating with optimal molecular features
Source: BMC Bioinformatics. 2023 Sep 12;24:338. doi: 10.1186/s12859-023-05455-1 (PMC10496404; doi:10.1186/s12859-023-05455-1)

**Prediction of the effects of small molecules on the gut microbiome** **using machine learning method integrating with optimal molecular features**

**SUPPLEMENTARY INFORMATION**

Binyou Wang^1,2^, Jianmin Guo^1^, Xiaofeng Liu^1^, Yang Yu^1,3^,

Jianming Wu ^1,3,4^* and Yiwei Wang ^1,3^*

^1^School of Basic Medical Sciences, Southwest Medical University, Luzhou 646000, China.

^2^School of Pharmacy, Southwest Medical University, Luzhou 646000, China.

^3^Key Laboratory of Medical Electrophysiology, Ministry of Education & Medical Electrophysiological Key Laboratory of Sichuan Province, Institute of Cardiovascular Research, Southwest Medical University, Luzhou 646000, China.

^4^Sichuan Key Medical Laboratory of New Drug Discovery and Druggability Evaluation; Luzhou Key Laboratory of Activity Screening and Druggability Evaluation for Chinese Materia Medica, School of Pharmacy, Southwest Medical University, Luzhou 646000, China.

*Correspondence: jianmingwu@swmu.edu.cn; wangyiwei0102@swmu.edu.cn

**Supplementary Methods**

**Support vector machine (SVM)**

SVM is a powerful supervised learning method that is widely used to solve classification problems. For linearly inseparable feature space, the kernel trick is needed to map the original feature space onto a new higher separable linear space. The basic objective of SVM algorithm is to define a decision boundary that optimally separates the two classes by identifying the optimal hyperplane.

***k*‐Nearest neighbor (*k*-NN)**

*k*-NN is a theoretically mature method and one of the simplest machine learning algorithms. It classifies an unknown sample with the most similar category of the k closest samples.

**Random forest (RF)**

RF is a classifier that contains multiple decision trees, and the output class is determined by the plurality of the output classes of individual trees. The training data were split into random subsets to fit individual trees. After training, prediction was made by aggregating (majority vote or averaging) the predictions of the individual trees.

**Naive Bayes (**NB**)**

NB is a simple probabilistic classifier based on the assumption of strong independence between features using Bayes' theorem.

**Gradient boosting machine (GBM)**

GBM belong to the decision-tree based ensemble method. The main idea of GBM is to build the next new base learner based on the gradient descent direction of the loss function of the previously established base learner, with the aim of integrating these base learners so that the overall loss function of the model keeps decreasing and the model keeps improving.

**Extreme gradient boosting (XGB)**

XGB is an extension of gradient boosting which uses multiple base learners. Each base learner is relatively simple to avoid overfitting, and the next learner learns the difference between the result of the previous base learner and the actual value, and keeps reducing the difference between the model value and the actual value by learning from multiple learners.

**Supplementary Formula**

The IG value was calculated as followings:

$$IG =Ent\left( D \right)- \sum_{V=0,1}^{V} \frac{D^{V}}{D}Ent\left( D^{V} \right)$$

$$Ent\left( D \right)=-\sum_{K=0,1}^{K} P_{k}\log_{2} P_{k}$$

where *V* represents the possible value of a fragment (0 or 1). *D* represents the number of all compounds, and $D^{V}$ represents the number of compounds with or without the fragment. $Ent\left( D \right)$ and $Ent\left( D^{V} \right)$ represent the information entropy and conditional entropy of compounds, respectively. *K* represents the classes of the compounds as 0 or 1 and $P_{k}$ is defined as the ratio of each class compounds.

The frequency of a fragment was calculated by the following equation:

$$Frequency of a fragment=\frac{N_{fragment\_P}N_{total}}{N_{fragment\_total}N_{P}}$$

where *N_fragment_p_* is the number of drugs containing the fragment in anticommensal compounds; *N_total_* is the total number of drugs; *N_fragment_total_* is the total number of drugs containing the fragment; and *N_p_* is the number of anticommensal compounds.

**Table S1.** Number of compounds in the training set and external validation set for 18 datasets.

| Group |  | Positives | Negatives |
| --- | --- | --- | --- |
| 1 | Training set | 305 | 640 |
|  | External validation set | 86 | 150 |
| 2 | Training set | 301 | 644 |
|  | External validation set | 90 | 146 |
| 3 | Training set | 333 | 612 |
|  | External validation set | 58 | 178 |
| 4 | Training set | 308 | 627 |
|  | External validation set | 83 | 153 |
| 5 | Training set | 337 | 608 |
|  | External validation set | 54 | 182 |
| 6 | Training set | 315 | 630 |
|  | External validation set | 76 | 160 |
| 7 | Training set | 317 | 628 |
|  | External validation set | 74 | 162 |
| 8 | Training set | 319 | 626 |
|  | External validation set | 72 | 164 |
| 9 | Training set | 320 | 625 |
|  | External validation set | 71 | 165 |
| 10 | Training set | 340 | 605 |
|  | External validation set | 51 | 185 |
| 11 | Training set | 315 | 630 |
|  | External validation set | 76 | 160 |
| 12 | Training set | 308 | 639 |
|  | External validation set | 83 | 153 |
| 13 | Training set | 322 | 623 |
|  | External validation set | 69 | 167 |
| 14 | Training set | 329 | 616 |
|  | External validation set | 62 | 174 |
| 15 | Training set | 322 | 623 |
|  | External validation set | 69 | 167 |
| 16 | Training set | 318 | 627 |
|  | External validation set | 73 | 163 |
| 17 | Training set | 319 | 626 |
|  | External validation set | 72 | 164 |
| 18 | Training set | 316 | 629 |
|  | External validation set | 75 | 161 |

**Table S2.** The sizes and pattern types of six molecular fingerprints used.

| **Fingerprints Type** | **Abbreviation** | **Pattern Type** | **Bit Size** |
| --- | --- | --- | --- |
| MDL Molecular Access | MACCS | Structural features | 166 |
| PubChem | PubChem | Structural features | 881 |
| Extended Connectivity(radius=4) | ECFP4-1 | Morgan fingerprints | 1024 |
| Extended Connectivity(radius=4) | ECFP4-2 | Morgan fingerprints | 2048 |
| Extended Connectivity(radius=6) | ECFP6-1 | Morgan fingerprints | 1024 |
| Extended Connectivity(radius=6) | ECFP6-2 | Morgan fingerprints | 2048 |

**Table S3.** The effect of standard scaler and min-max scaler on F1-scroe in five-fold cross-validation.

| **Group** | **Standard scaler** | **Min-max scaler** |
| --- | --- | --- |
| 1 | 0.729±0.011 | 0.734±0.014 |
| 2 | 0.700±0.013 | 0.703±0.008 |
| 3 | 0.721±0.014 | 0.727±0.009 |
| 4 | 0.697±0.011 | 0.696±0.009 |
| 5 | 0.735±0.011 | 0.739±0.013 |
| 6 | 0.717±0.007 | 0.721±0.010 |
| 7 | 0.704±0.012 | 0.712±0.014 |
| 8 | 0.744±0.011 | 0.747±0.013 |
| 9 | 0.703±0.010 | 0.708±0.009 |
| 10 | 0.739±0.011 | 0.739±0.011 |
| 11 | 0.726±0.013 | 0.736±0.014 |
| 12 | 0.711±0.013 | 0.716±0.007 |
| 13 | 0.732±0.008 | 0.734±0.006 |
| 14 | 0.730±0.010 | 0.742±0.012 |
| 15 | 0.728±0.010 | 0.732±0.007 |
| 16 | 0.721±0.010 | 0.729±0.009 |
| 17 | 0.720±0.014 | 0.728±0.006 |
| 18 | 0.716±0.009 | 0.715±0.009 |

**Table S4.** Range of parameters to be adjusted in five-fold cross-validation.

| **Methods** | **Hyperparameters** | **Values range** |
| --- | --- | --- |
| SVM | penalty parameter C  kernel parameter gamma | {1000,5000,10000,50000}  {0.001,0.005,0.0001,0.0005} |
| RF | number of estimators | {100-1000} |
| *k*-NN  GBM | number of neighbors  weighting schemes  number of estimators  learning rate | {1, 3, 5, 7, 9}  {uniform, distance}  {500-1000}  {0.1-0.3} |
| XGB | maximum depth of tree  minimum sum of instance weight in child | {3,5,8,10}  {1, 3, 5} |

**Table S5 and Table S6 can be found in Additional file 2.**

**Table S7.** The F1-score values of the top based models the optimal XGB models based on the combined features, fingerprints and descriptors for each training set.

| **Group** | **Combined features** | **Fingerprints** | **Descriptors** |
| --- | --- | --- | --- |
| 1 | 0.734 ± 0.014 | 0.680 ± 0.010 | 0.696 ± 0.011 |
| 2 | 0.703 ± 0.008 | 0.648 ± 0.018 | 0.639 ± 0.012 |
| 3 | 0.727 ± 0.009 | 0.700 ± 0.014 | 0.711 ± 0.014 |
| 4 | 0.696 ± 0.009 | 0.667 ± 0.010 | 0.674 ± 0.012 |
| 5 | 0.739 ± 0.013 | 0.692 ± 0.014 | 0.704 ± 0.010 |
| 6 | 0.721 ± 0.010 | 0.679 ± 0.013 | 0.696 ± 0.010 |
| 7 | 0.712 ± 0.014 | 0.673 ± 0.014 | 0.690 ± 0.009 |
| 8 | 0.747 ± 0.013 | 0.703 ± 0.013 | 0.698 ± 0.007 |
| 9 | 0.708 ± 0.009 | 0.675 ± 0.010 | 0.684 ± 0.011 |
| 10 | 0.739 ± 0.011 | 0.716 ± 0.015 | 0.720 ± 0.008 |
| 11 | 0.738 ± 0.014 | 0.705 ± 0.009 | 0.708 ± 0.012 |
| 12 | 0.716 ± 0.007 | 0.700 ± 0.012 | 0.711 ± 0.009 |
| 13 | 0.734 ± 0.006 | 0.704 ± 0.012 | 0.698 ± 0.011 |
| 14 | 0.742 ± 0.012 | 0.689 ± 0.012 | 0.713 ± 0.015 |
| 15 | 0.732 ± 0.007 | 0.688 ± 0.014 | 0.705 ± 0.007 |
| 16 | 0.729 ± 0.009 | 0.676 ± 0.009 | 0.687 ± 0.010 |
| 17 | 0.728 ± 0.006 | 0.691 ± 0.011 | 0.711 ± 0.010 |
| 18 | 0.741 ± 0.009 | 0.685 ± 0.012 | 0.707 ± 0.011 |

**Table S8.** The five-fold cross-validation results of the 18 optimal XGB-MACCS+13MD models.

| **Group** | **SE (%)** | **SP (%)** | **ACC (%)** | **MCC** | **AUC** | **F1-score** |
| --- | --- | --- | --- | --- | --- | --- |
| 1 | 68.2±1.4 | 91.7±0.6 | 84.1±0.7 | 0.627±0.019 | 0.800±0.009 | 0.734±0.014 |
| 2 | 63.9±0.9 | 91.9±0.5 | 83.0±0.4 | 0.594±0.010 | 0.779±0.005 | 0.703±0.008 |
| 3 | 68.5±1.1 | 89.4±0.4 | 82.0±0.5 | 0.598±0.012 | 0.789±0.006 | 0.727±0.009 |
| 4 | 63.8±0.9 | 90.8±0.7 | 82.0±0.6 | 0.576±0.014 | 0.773±0.006 | 0.696±0.009 |
| 5 | 68.5±1.8 | 91.0±0.4 | 82.9±0.7 | 0.620±0.017 | 0.797±0.010 | 0.739±0.013 |
| 6 | 66.1±1.1 | 91.6±0.8 | 83.0±0.6 | 0.607±0.014 | 0.788±0.007 | 0.721±0.010 |
| 7 | 66.2±1.3 | 90.2±0.8 | 82.1±0.9 | 0.588±0.020 | 0.782±0.010 | 0.712±0.014 |
| 8 | 69.0±1.4 | 92.2±0.7 | 84.3±0.8 | 0.641±0.018 | 0.806±0.007 | 0.747±0.013 |
| 9 | 66.1±1.5 | 89.7±0.7 | 81.7±0.5 | 0.581±0.013 | 0.779±0.007 | 0.708±0.009 |
| 10 | 69.2±1.2 | 90.0±0.7 | 82.5±0.7 | 0.613±0.017 | 0.796±0.008 | 0.739±0.011 |
| 11 | 69.2±1.7 | 90.7±0.7 | 83.5±0.8 | 0.621±0.019 | 0.800±0.010 | 0.736±0.014 |
| 12 | 66.7±0.9 | 90.9±0.6 | 82.9±0.4 | 0.601±0.010 | 0.788±0.005 | 0.716±0.007 |
| 13 | 68.4±0.8 | 90.9±0.6 | 83.2±0.4 | 0.617±0.009 | 0.797±0.004 | 0.734±0.006 |
| 14 | 69.6±1.8 | 90.5±0.6 | 83.2±0.7 | 0.622±0.015 | 0.801±0.009 | 0.742±0.012 |
| 15 | 67.6±0.9 | 91.3±0.7 | 83.2±0.5 | 0.616±0.010 | 0.794±0.007 | 0.732±0.005 |
| 16 | 68.3±1.2 | 90.5±0.6 | 83.0±0.5 | 0.611±0.011 | 0.794±0.006 | 0.729±0.009 |
| 17 | 68.7±1.0 | 90.0±0.8 | 82.8±0.5 | 0.607±0.010 | 0.793±0.004 | 0.728±0.006 |
| 18 | 66.1±1.1 | 90.8±0.5 | 82.4±0.5 | 0.596±0.013 | 0.784±.006 | 0.715±0.009 |

**Table S9.** The statistical results for the 18 groups of datasets given by best models based on four machine learning methods with 13MD+MACCS.

| **Group** | **Model** | **SE (%)** | **SP (%)** | **ACC (%)** | **MCC** | **AUC** | **F1-score** |
| --- | --- | --- | --- | --- | --- | --- | --- |
| 1 | GBM-13MD+MACCS | 67.9±2.3 | 89.9±0.6 | 82.8±0.8 | 0.598±0.021 | 0.789±0.012 | 0.717±0.016 |
| 2 | GBM -13MD+MACCS | 65.3±2.4 | 89.1±0.8 | 81.5±0.9 | 0.562±0.024 | 0.772±0.013 | 0.690±0.019 |
| 3 | RF-13MD+MACCS | 64.8±1.1 | 91.7±0.7 | 82.2±0.6 | 0.601±0.014 | 0.783±0.007 | 0.718±0.009 |
| 4 | RF-13MD+MACCS | 60.6±1.1 | 93.0±0.4 | 82.4±0.4 | 0.585±0.011 | 0.768±0.006 | 0.690±0.009 |
| 5 | RF-13MD+MACCS | 65.3±1.1 | 91.7±0.5 | 82.4±0.6 | 0.605±0.014 | 0.785±0.007 | 0.723±0.010 |
| 6 | GBM-13MD+MACCS | 66.5±1.5 | 89.3±0.8 | 81.6±0.6 | 0.577±0.012 | 0.779±0.007 | 0.705±0.010 |
| 7 | RF-13MD+MACCS | 63.6±1.4 | 91.5±0.4 | 82.0±0.6 | 0.586±0.015 | 0.775±0.008 | 0.703±0.011 |
| 8 | RF-13MD+MACCS | 67.3±1.7 | 92.5±0.4 | 83.9±0.7 | 0.632±0.017 | 0.799±0.009 | 0.738±0.013 |
| 9 | RF-13MD+MACCS | 62.4±1.3 | 92.1±0.4 | 82.0±0.5 | 0.585±0.012 | 0.773±0.06 | 0.700±0.010 |
| 10 | RF-13MD+MACCS | 66.2±1.4 | 91.5±0.6 | 82.3±0.6 | 0.608±0.014 | 0.788±0.011 | 0.728±0.008 |
| 11 | GBM-13MD+MACCS | 70.3±1.6 | 89.1±1.5 | 82.8±1.2 | 0.607±0.027 | 0.797±0.012 | 0.730±0.017 |
| 12 | RF-13MD+MACCS | 65.9±1.4 | 91.9±0.4 | 83.4±0.5 | 0.610±0.013 | 0.789±0.007 | 0.720±0.011 |
| 13 | RF-13MD+MACCS | 65.1±0.9 | 91.8±0.8 | 82.6±0.6 | 0.603±0.013 | 0.784±0.005 | 0.717±0.008 |
| 14 | RF-13MD+MACCS | 66.8±1.0 | 91.8±0.7 | 83.1±0.5 | 0.618±0.013 | 0.793±0.006 | 0.732±0.008 |
| 15 | RF-13MD+MACCS | 64.3±1.1 | 93.1±0.4 | 83.2±0.5 | 0.617±0.011 | 0.787±0.006 | 0.721±0.009 |
| 16 | RF-13MD+MACCS | 64.9±0.7 | 92.6±0.6 | 83.2±0.5 | 0.614±0.012 | 0.787±0.004 | 0.721±0.008 |
| 17 | RF-13MD+MACCS | 65.5±1.1 | 91.8±0.6 | 82.8±0.4 | 0.605±0.009 | 0.786±0.007 | 0.719±0.005 |
| 18 | GBM-13MD+MACCS | 67.6±1.8 | 89.0±0.9 | 81.8±0.7 | 0.583±0.016 | 0.783±0.008 | 0.711±0.012 |
| consensus | / | 65.6±2.2 | 91.3±1.4 | 82.6±0.7 | 0.600±0.017 | 0.784±0.008 | 0.716±0.013 |

**Figure S1**. Misclassified anticommensal compounds of the consensus model on the external validation set.


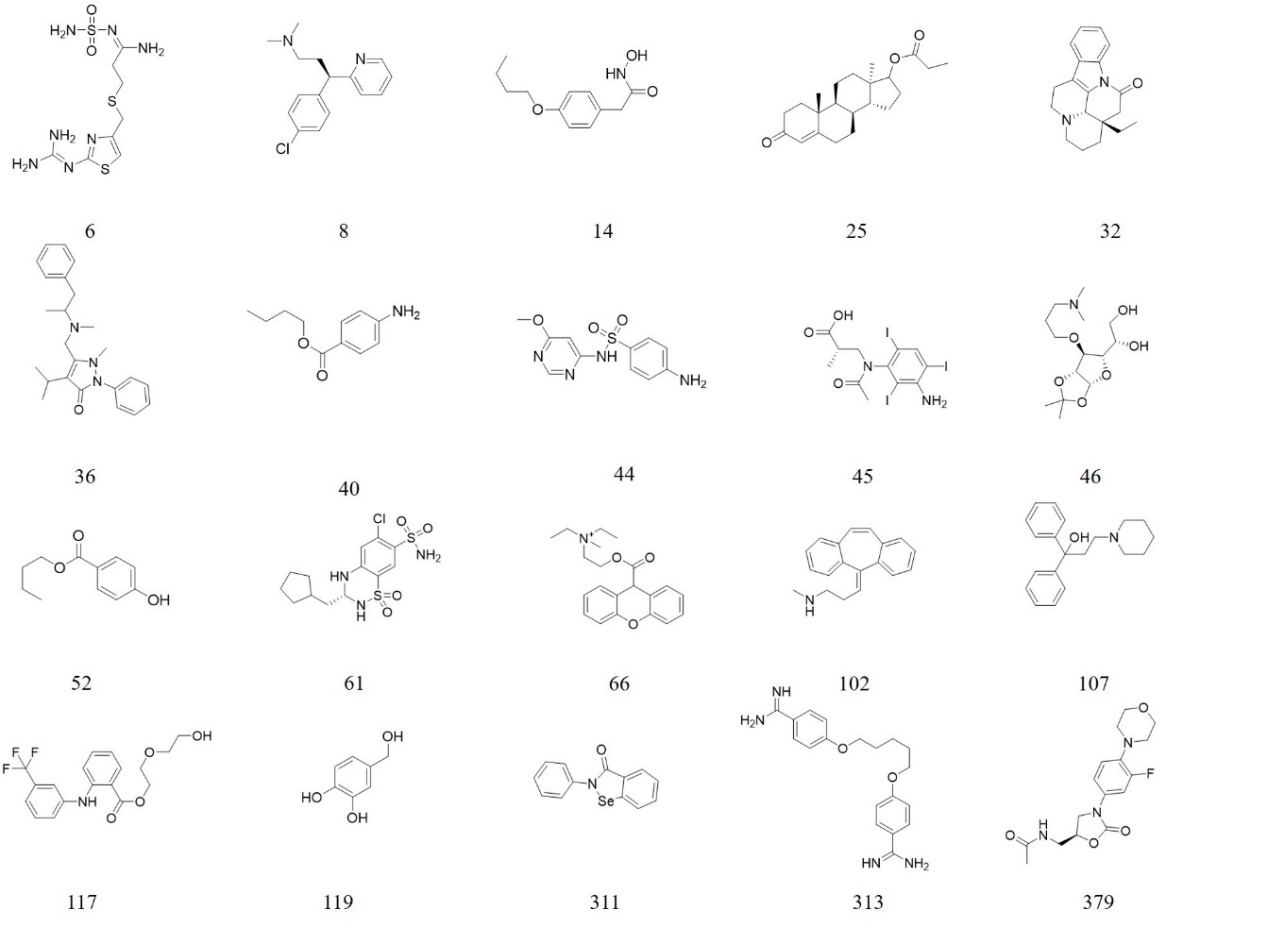

Supplement: Supplementary file 1 — Additional file 1. Detailed information of supplementary methods, supplementary formula, supplementary tables S1–S4 and supplementary tables S7–S9, supplementary figure S1. [file 12859_2023_5455_MOESM1_ESM.docx]
